# Multiplicity of *Salmonella* entry mechanisms, a new paradigm for *Salmonella* pathogenesis

**DOI:** 10.1002/mbo3.28

**Published:** 2012-06-18

**Authors:** P Velge, A Wiedemann, M Rosselin, N Abed, Z Boumart, A M Chaussé, O Grépinet, F Namdari, S M Roche, A Rossignol, I Virlogeux-Payant

**Affiliations:** 1 INRA, UMR1282 Infectiologie et Santé PubliqueF-37380, Nouzilly, France; 2 Université François Rabelais de Tours, UMR1282 Infectiologie et Santé PubliqueF-37000, Tours, France; 3 Agence Nationale de Sécurité Sanitaire de l'alimentation, de l'environnement et du travail, Laboratoire de Ploufragan-Plouzané, Unité Hygiène et Qualité des Produits Avicoles et Porcins22440, Ploufragan, France

**Keywords:** Adhesion, invasin, invasion, *Salmonella*, Trigger, type III secretion system, Zipper

## Abstract

The *Salmonella enterica* species includes about 2600 diverse serotypes, most of which cause a wide range of food- and water-borne diseases ranging from self-limiting gastroenteritis to typhoid fever in both humans and animals. Moreover, some serotypes are restricted to a few animal species, whereas other serotypes are able to infect plants as well as cold- and warm-blooded animals. An essential feature of the pathogenicity of *Salmonella* is its capacity to cross a number of barriers requiring invasion of a large variety of phagocytic and nonphagocytic cells. The aim of this review is to describe the different entry pathways used by *Salmonella* serotypes to enter different nonphagocytic cell types. Until recently, it was accepted that *Salmonella* invasion of eukaryotic cells required only the type III secretion system (T3SS) encoded by the *Salmonella* pathogenicity island-1. However, recent evidence shows that *Salmonella* can cause infection in a T3SS-1-independent manner. Currently, two outer membrane proteins Rck and PagN have been clearly identified as *Salmonella* invasins. As Rck mediates a Zipper-like entry mechanism, *Salmonella* is therefore the first bacterium shown to be able to induce both Zipper and Trigger mechanisms to invade host cells. In addition to these known entry pathways, recent data have shown that unknown entry routes could be used according to the serotype, the host and the cell type considered, inducing either Zipper-like or Trigger-like entry processes. The new paradigm presented here should change our classic view of *Salmonella* pathogenicity. It could also modify our understanding of the mechanisms leading to the different *Salmonella*-induced diseases and to *Salmonella-*host specificity.

## *Salmonella* and Salmonelloses

### The bacteria and diseases

*Salmonella* is a member of the Enterobacteriaceae family, a large group of Gram-negative, facultative anaerobic and nonspore-forming bacilli. The genus *Salmonella* consists of only two species, *Salmonella bongori* and *Salmonella enterica,* and the latter is divided into six subspecies: *enterica*, *salamae*, *arizonae*, *diarizonae*, *houtenae*, and *indica* (Guibourdenche et al. 2010). The agglutinating properties of the somatic O, flagellar H, and capsular Vi antigens are used to differentiate more than 2600 serologically distinct *Salmonella* (Guibourdenche et al. 2010). Strains belonging to *S. enterica* subsp. *enterica* cause approximately 99% of *Salmonella* infections in humans and warm-blooded animals (McClelland et al. 2001). Moreover, this subspecies is able to infect plants and numerous international outbreaks of *S. enterica* have been linked to plant contamination (Pezzoli et al. 2007; Nygard et al. 2008). Serotypes in other subspecies are usually isolated from cold-blooded animals and the environment, but rarely from humans (Uzzau et al. 2000). *Salmonella* nomenclature is now based on the name of serotypes belonging to subspecies. For example, *Salmonella enterica* subsp. *enterica* serotype Typhimurium is shortened to *Salmonella* Typhimurium (Brenner et al. 2000).

From a clinical perspective, *Salmonella* serotypes may be broadly grouped on the basis of host range and disease outcomes (Uzzau et al. 2000). Host-specific serotypes are associated with severe systemic disease in adults of a single species, which may seldom involve diarrhea (e.g., *Salmonella* Typhi, *Salmonella* Gallinarum). For example, *S*. Typhi is a human-specific pathogen causing a septicaemic typhoid syndrome (enteric fever). *S*. Gallinarum has a host range restricted to birds and causes a severe systemic disease called fowl typhoid (Shivaprasad 2000). Host-restricted serotypes are primarily associated with systemic disease in few hosts (e.g., *Salmonella* Dublin in cattle, *Salmonella* Choleraesuis in pigs and humans), but may cause disease in a limited number of other species (Chiu et al. 2004). Broad host range serotypes, such as *S*. Typhimurium and *S*. Enteritidis cause the majority of human gastrointestinal salmonelloses (Velge et al. 2005). They are able to infect, among many other animal species, domestic livestock and fowl worldwide, resulting in a spectrum of outcomes ranging from severe systemic disease to asymptomatic carriage. For example, in contrast to *S*. Gallinarum, which induces a systemic infection only in fowl, *S*. Enteritidis and *S*. Typhimurium generate a subclinical intestinal infection in poultry after a short systemic infection, and infected hens can become chronic carriers and lay contaminated eggs (Shivaprasad 2000; Velge et al. 2005). In humans and cattle, infection with these broad host range serotypes manifests as enterocolitis, usually limited to the gastrointestinal tract and rarely spreads to systemic organs (Stevens et al. 2009). In susceptible mice, these serotypes cross the gut epithelium efficiently, colonize the spleen and the liver, and are consequently responsible for a typhoid-like disease (Santos et al. 2001). Moreover, even within the same serotype, different strains isolated from animals or humans exhibited different virulence levels (Heithoff et al. 2008), and host-restricted variants exist, such as pigeon-associated *S*. Typhimurium definitive type (DT)-2 and DT99 strains (Rabsch et al. 2002). Finally, for the same host, the different *Salmonella* serotypes can induce different pathologies. For example, oral inoculation of weaned calves with *S*. Dublin produces severe systemic infection, whereas *S*. Gallinarum is avirulent and *S*. Typhimurium elicits acute enteritis (Paulin et al. 2002). Pathogenesis is not only influenced by the dose and route of inoculation but also by the genetic background and immune status of the host (Calenge et al. 2009, 2010). In summary, no other bacterial pathogen belonging to a single species shows such a remarkable ability to infect different hosts and induce so many different diseases.

### Host invasion pathways

Despite the availability of complete genome sequences of isolates representing several serotypes, the molecular mechanisms underlying *Salmonella* colonization, pathogenesis, and transmission have been described mainly in rodents, and thus little is known about these mechanisms in farm animals and humans.

Host infections are usually initiated by ingestion of contaminated food or water followed by the passage of the bacteria from the stomach to the intestine. There, the bacteria adhere to and enter the cells lining the intestinal epithelium. Passage of the bacteria through the intestinal wall is believed to be initiated by transcytosis, that is, invasion of either enterocytes or M cells at the apical side, migration to the basolateral side, and exocytosis into the interstitial space of the lamina propria (Takeuchi 1967; Clark et al. 1994; Muller et al. 2012). Direct capture by CD18^+^ phagocytes and CD11b^+^, CD11c^+^, CX_3_CR1^high^ phagocytes has also been observed (Vazquez-Torres et al. 1999; Muller et al. 2012). Within the lamina propria, *S*. Typhimurium is taken up randomly by the different phagocytes (macrophages, dendritic cells, and polymorphonuclear cells) and disseminates rapidly through efferent lymph in mesenteric lymph nodes and through the blood stream in spleen and liver (Salcedo et al. 2001). However, different behaviors have been observed depending on the serotype and the host. In cattle, *S*. Dublin transits rapidly through the epithelial layer and associates with MHC class II-positive cells in the lamina propria. These bacteria are predominantly extracellular within efferent lymph, but it remains unclear how they arrive in draining nodes or escape into an extracellular niche in this organ (Pullinger et al. 2007). *S*. Typhi, like *S*. Typhimurium, is capable of entering the murine intestinal epithelium via M cells. However, unlike *S*. Typhimurium, it does not destroy the epithelium and is cleared from the Peyer's patches soon after M-cell entry (Pascopella et al. 1995).

### Cell invasion pathways

**Figure 1:**
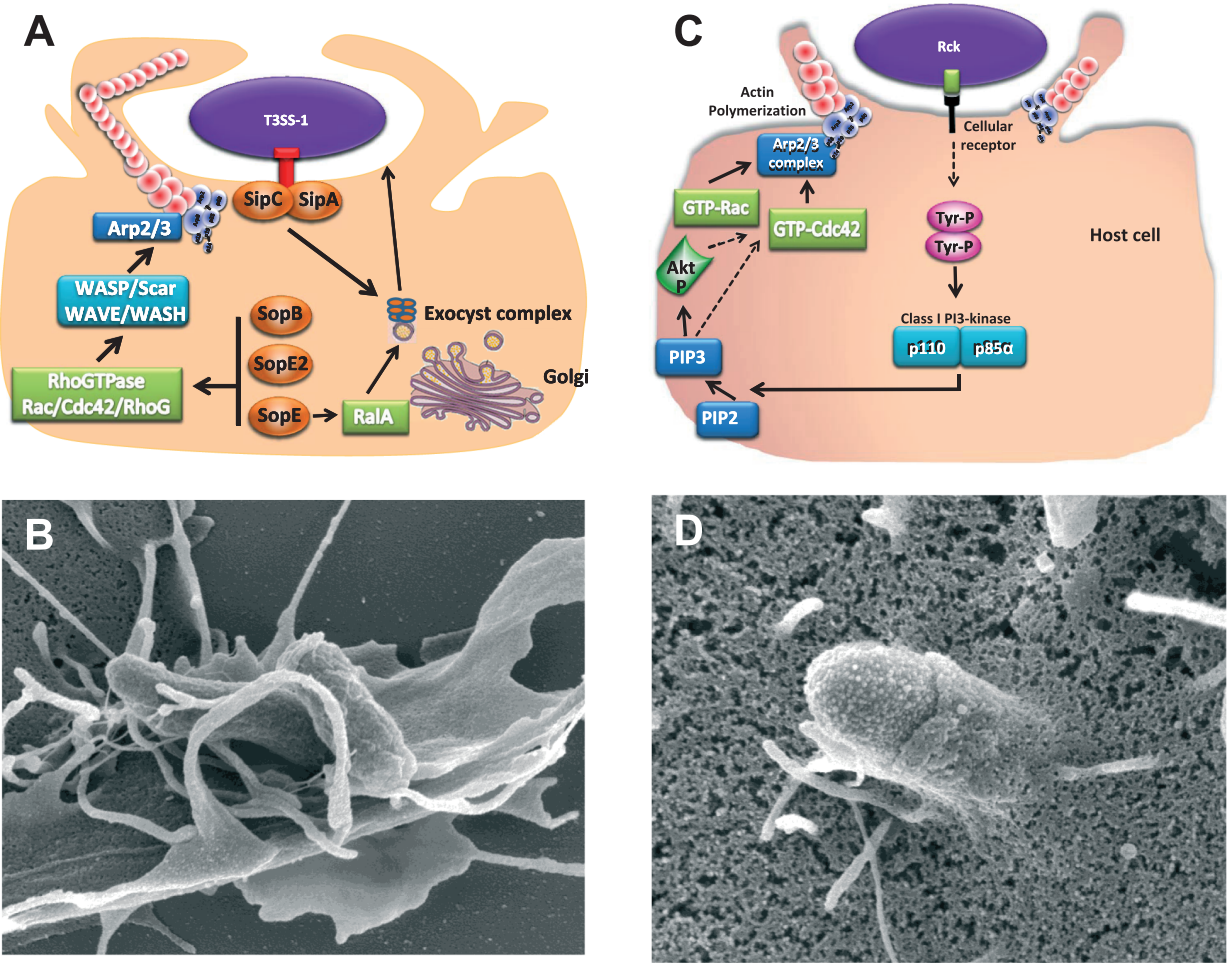
Trigger and Zipper mechanisms used by *Salmonella* to enter cells. (A) Schematic representation of the Trigger mechanism: Using a type III secretion system (T3SS), *Salmonella* bacterial effectors (SipA, SipC, SopB, SopE, SopE2) are directly injected into eukaryotic cells. SopE, SopE2, and SopB activate the RhoGTPases Rac/Cdc42/RhoG to allow actin cytoskeleton remodeling via cellular proteins, such as WASP/Scar/WAVE/WASH, which activate the Arp2/3 complex. In contrast, SipA and SipC bind directly to actin. To induce the formation of membrane ruffles and internalization, the recruitment of the exocyst complex is required and is manipulated by SipC and by SopE via the Ras-related protein RalA. (B) Scanning electron microscopy of *Salmonella* entering into cells via a Trigger mechanism, which is characterized by the apparition of large membrane ruffles at the bacterial entry site. (C) Schematic representation of the Zipper mechanism: the Rck invasin expressed on *Salmonella* outer membrane interacts with its receptor on the host cell membrane, leading to phosphorylation of at least one tyrosine kinase. Activation of the class I PI 3-kinase induces PI (3,4,5)P3 formation, participating to Akt activation. The activation of the GTPase Rac1, downstream of the Akt/PI 3-kinase activation, and the GTPase Cdc42 trigger actin polymerization via the Arp2/3 nucleator complex. The mechanism controlling Cdc42 during Rck-induced signaling pathway is still unknown. Dotted arrows represent possible signaling events and/or interactions. (D) Scanning electron microscopy of *Salmonella* entering into cells via a Zipper mechanism, which is characterized by weak membrane rearrangements.

The reasons why some *Salmonella* serotypes are confined to the intestine while others translocate to distal organs remain unclear. An essential feature of the pathogenicity of *Salmonella* is its interaction with phagocytic and nonphagocytic cells, and *Salmonella* entry into host cells is known to be critical for bacterial survival and establishment of disease in a host. In general, intracellular bacterial pathogens enter nonphagocytic eukaryotic cells via two mechanisms, which are initially differentiated according to morphological criteria based on membrane remodeling. The “Trigger” mechanism involves dramatic cytoskeletal rearrangements known as “membrane ruffles” (Fig. 1A and B). In contrast, in the “Zipper” mechanism, or “receptor-mediated entry,” the invading bacteria are tightly bound to the host cell membrane, and only minor cytoskeletal protein rearrangements are initiated by specific contact between bacterial ligands (invasin) and host cell surface receptors (Fig. 1C and D). An important mechanistic difference between the Trigger and Zipper modes of entry is that the former is triggered from “inside” via the action of bacterial effectors delivered by secretion systems, whereas the latter is promoted from “outside” through activation of host cell receptors. However, in both cases, bacteria hijack the cell's physiological processes through the modulation of existing cell signaling cascades. It has recently been reported that *Salmonella* is the first bacteria shown to be able to enter cells using both these mechanisms (Rosselin et al. 2010). An emerging idea is that *Salmonella* strains can enter nonphagocytic cells by multiple pathways involving a Trigger or a Zipper mechanism. This is in contrast to the prevailing paradigm of *Salmonella* pathogenesis asserting that the *Salmonella* pathogenicity island-1 type III secretion system (SPI-1 T3SS or T3SS-1) is essential for bacterial invasion of host cells. The T3SS-1 and other cell invasion mechanisms are described below.

## Role of T3SS-1 in Cell Invasion

### The SPI-1 encodes the T3SS-1

Pathogenicity islands (PAIs) are genetic elements integrated into the core genome of bacteria, which confer a virulence phenotype on the bacteria that have acquired them (Groisman and Ochman 1996; Shames et al. 2009). Currently, 21 different *Salmonella* pathogenicity islands (SPIs) have been identified in *Salmonella* (Blondel et al. 2009). However, many of the identified PAI-encoded genes have only predicted putative functions with no clear role in *Salmonella* pathogenesis (Blondel et al. 2010). The SPI-1 locus is a 40-kb chromosomal island, which carries among others all the genes required for the biosynthesis of a functional T3SS apparatus, a number of effector proteins and their chaperones, and some regulatory proteins (Galan and Curtiss 1989; Galan and Collmer 1999). A T3SS is a multi-subunit protein complex capable of injecting effectors directly from bacterial cytoplasm into the host cell cytosol. These effectors modulate cellular processes to the benefit of the pathogen. The T3SS consists of a needle complex and an export apparatus allowing the secreted proteins to pass through the bacterial inner and outer membranes, and of a translocon which creates a pore in the host cell membrane. The T3SS-1, encoded by SPI-1, is among the best characterized of all *Salmonella* virulence factors and triggers entry of *Salmonella* in a wide range of eukaryotic cells.

Of all SPIs reported in *Salmonella* only SPI-1, SPI-4, and SPI-9 are present in both *Salmonella bongori* and *S. enterica* species suggesting that they were acquired by *Salmonella* at the beginning of the evolution of *Salmonella* and prior to speciation. Moreover, 10 of the 15 known T3SS-1 translocated effectors are almost entirely conserved in *S. enterica* and *S. bongori*. Genes encoding effectors are even found at the same genomic loci in *S. bongori* as they are in *S. enterica*, that is, carried on SPI-1 itself or on SPI-5 or at identical sites in the chromosomal backbone (Fookes et al. 2011). The use of microarrays to identify the virulence gene profiles in 24 *Salmonella* strains of different serotypes from food and/or food animal environment showed that nearly 58% of the virulence-associated genes tested were present in all *Salmonella* strains tested. In general, genes belonging to *inv, prg, sic, sip*, or *spa* families were detected in more than 90% of the isolates, whereas the *iacP, avrA, invH, sopB*, or *sopE* genes were detected in 40–80% of the isolates (Zou et al. 2011). Moreover, an epidemiological analysis suggested that *sopE,* which is encoded by a temperate bacteriophage, appears to be associated with epidemic strains (Hopkins and Threlfall 2004), whereas *sopE2* is present in all *Salmonella* strains tested (Bakshi et al. 2000).

### Role of T3SS-1 in cell invasion

Inside the host, after entering the lumen of the small intestine, *Salmonella* sense the environment (pH, oxygen tension, osmolarity, etc.), enabling T3SS-1 genes to be expressed and subsequently the secretion apparatus to be assembled at the bacterial membrane (Galan and Collmer 1999; Rosselin et al. 2012). Host cell invasion is initiated by pathogen binding to the host cell surface, which activates the insertion of the translocon into the host cell membrane through its affinity for cholesterol (Hayward et al. 2005). This bacteria-cell contact allows translocation of effectors into the host cell. This translocation is precisely coordinated ensuring that bacterial proteins engage in a coherent order through a cytoplasmic sorting platform, which ensures secretion of the translocases (SipB, SipC, and SipD) before the effectors. The sequential loading on this platform may be facilitated by the different affinities of the T3SS-chaperones, ensuring the hierarchy in type III effector secretion (Lara-Tejero et al. 2011). Cell entry is characterized by profuse rearrangements of the actin cytoskeleton at the site of bacteria–host-cell contact, which envelop external bacteria and internalize them into membrane-bound vacuoles. Currently, it seems difficult to know whether the entry process mediated by the T3SS-1 is the same for all cell types (phagocytic and nonphagocytic cells), mainly because no experiment has been designed to compare the entry processes between cell lines. Only one experiment was performed to compare the transcriptome of a *S*. Typhimurium strain within a phagocytic and a nonphagocytic cell line (Hautefort et al. 2008). This study especially showed that SPI-1- and flagella-related genes were expressed inside epithelial cells at later stages of the infection than in macrophage-like cells. At least 15 effectors can be translocated into the host cell by T3SS-1 to induce bacterial entry (reviewed in McGhie et al. 2009). Among them, five major *Salmonella* effectors, each being able to manipulate the cytoskeletal machinery within the host, are known to drive engulfment (SopE, SopE2, SopB, SipA, and SipC) (Fig. 1A).

SopE, SopE2, and SopB target the RhoGTPase switch and promote activation of the Rho family members Cdc42 and Rac, leading to activation of N-WASP- and Scar/WAVE-Arp2/Arp3 (Arp2/3) complexes which trigger actin remodeling. Despite their differing biochemical activities, these effectors have key redundant roles during internalization. Indeed, *Salmonella* strains lacking just one of these effectors display only a modest reduction in entry, whereas a triple Δ*sopE/E2/B* mutant is completely abrogated for cytoskeletal remodeling and entry (Zhou et al. 2001). SopE and SopE2 function as guanine exchange factors (GEFs) and directly catalyze GTPase activation. SopB is an inositol phosphatase that acts on host cell membrane phospholipids and thus functions indirectly by activating endogenous eukaryotic SH-3-containing GEF (SGEF), an exchange factor for the Rho-family GTPase: RhoG (Patel and Galan 2006). Recent data also showed that SopB constitutes an important regulator of an additional entry pathway which activates RhoA, the Rho kinase, and myosin II leading to stress fiber formation and contractility (Hanisch et al. 2011).

SipA and SipC can engage actin directly controlling and localizing actin polymerization at bacterial attachment site. SipC possesses distinct C- and N-terminal domains which are able to nucleate filamentous actin (F-actin) and promote F-actin bundling (Myeni and Zhou 2010). Chang et al. (2005) demonstrated that the effector translocation function of SipC is dissociable from the actin-nucleating function. In vitro*,* SipA stimulates these SipC activities and can stabilize F-actin by directly antagonizing the action within the cell of depolymerizing factors (Zhou et al. 1999), such as ADF/cofilin and gelsolin (McGhie et al. 2004). However, recent single molecule imaging studies have questioned the latter finding (Popp et al. 2008). SipA also plays an important role in induction of invasion-competent membrane ruffles in synergy with the other major effectors (Perrett and Jepson 2009). Invasion efficiency is also increased by promoting localized membrane expansion directly through SipC-dependent recruitment of the exocyst and indirectly via SopE-dependent activation of RalA (Braun and Brumell 2010; Nichols and Casanova 2010). Nichols and Casanova (2010) have proposed a role for the exocyst in delivering vesicles to the site of bacterial entry to provide additional membranes to allow the extension and ruffling of the plasma membrane necessary to promote invasion.

The actin remodeling events initiated by these five major effectors which undergo profuse membrane ruffling after 10–30 min of contact are transient and typically reversed 2–3 h postentry to display a normal actin cytoskeleton, despite the presence of a large number of intracellular bacteria (Kubori and Galan 2003). Remarkably, *Salmonella* actively helps the host cell to regain its normal cellular architecture through the action of SptP, a SPI-1 effector with GAP activity that returns Cdc42 and Rac1 to the nonactivated state. The interplay between the bacterial effectors acting as GEFs and GAPs (SopE/SptP) is based on different half-lives of these proteins following translocation (Kubori and Galan 2003). SptP shows a higher resistance to the ubiquitin-proteasome system, and downregulates Cdc42 and Rac1 once SopE is degraded.

These effectors involved in T3SS-1-dependent entry also have profound effects on later processes, such as membrane trafficking, cell division, apoptosis, bacterial killing, cytokine and chemokine production, and antigen presentation (reviewed in Santos et al. 2009). It is noteworthy that several T3SS-1 effectors also mediate the disruption of tight junctions and thus impair intestinal barrier integrity (Boyle et al. 2006). After bacterial entry, SopB also plays a role in *Salmonella*-containing vacuole (SCV) biogenesis, sealing, and trafficking. Recently, it has been reported that ubiquitination downregulates SopB activity at the plasma membrane, but prolongs retention of SopB on the induced vesicles enriched in phospho-inositide (3)P (Knodler et al. 2009; Patel et al. 2009). The SCV initially acquires the early endosome markers, which are sequentially replaced by the late endosome and lysosome markers. However, the SCV does not fuse directly with the lysosomes, thereby avoiding *Salmonella* destruction, partly due to the phospho-inositide phosphatase activity of SopB (Rudge et al. 2004). By reducing the local concentration of PI(4,5)P2, SopB cooperates with SopD to destabilize cytoskeleton-plasma membrane interactions and to reduce membrane rigidity, promoting the fission and the sealing of the future SCV (Bakowski et al. 2007).

As infection progresses, *Salmonella* uses a second type III secretion system, the T3SS-2, encoded by the SPI-2 which delivers additional effector proteins through the SCV membrane allowing bacterial survival and replication (reviewed in Malik-Kale et al. 2011). Nevertheless, it has been demonstrated that SPI-1 T3SS effectors may be involved in vacuole biogenesis and intracellular survival, functions which were previously attributed solely to the actions of SPI-2 T3SS effectors (Steele-Mortimer et al. 2002).

## Role of T3SS-1 in *Salmonella* Pathogenesis

### Involvement of the T3SS-1 in host infection

Based on studies on the cellular and molecular mechanisms of *S*. Typhimurium infection, SPI-1 is considered to be essential for the invasion of animal cells by *Salmonella*, and SPI-2 is required for intracellular proliferation and survival. In vivo experiments have been performed in different animal species to investigate the role of SPI-1 T3SS during the course of *Salmonella* infection.

In a mouse model of systemic lethal infection, it was observed that *S*. Typhimurium mutants unable to assemble functional T3SS-1 were recovered from intestinal contents and systemic sites at a lower level than the wild-type strain after oral but not after intraperitoneal inoculation (Galan and Curtiss 1989). The T3SS-1 is also involved in a mouse model of *Salmonella*-induced colitis based on the pretreatment of animals with streptomycin. It was demonstrated that the SPI-1 effectors SipA, SopE, and SopE2, in addition to flagella and chemotaxis, were required to induce intestinal inflammation and significant histopathological changes (Hapfelmeier et al. 2004; Stecher et al. 2004).

Within minutes of injecting *Salmonella* into ligated ileal loops in calves, *Salmonella* can be seen to invade both M cells and enterocytes (Frost et al. 1997). This is in marked contrast to what is seen in mice where the major portal of entry appears to be through M cells (Jones et al. 1994). Different in vivo studies have demonstrated that mutations in T3SS-1 decrease enterocyte invasion and abolish induction of fluid secretion and the recruitment of polymorphonuclear cells in bovine ligated ileal loops (Galyov et al. 1997).

Similarly, *S*. Typhimurium SPI-1 mutants are impaired in their ability to colonize the porcine gut in a ligated intestinal loop model (Boyen et al. 2006). The involvement of SPI-1 and not of other SPIs in proinflammatory signaling and heterophil infiltration in the intestine has also been demonstrated in chicks infected with *S*. Enteritidis (Rychlik et al. 2009). In this model, the role of SPI-1 and SPI-2 in the liver and spleen colonization has also been shown. Mutants lacking all the five major pathogenicity islands (SPI 1-5), but expressing only SPI-1 or SPI-2 have a medium virulence compared with the wild-type *S*. Enteritidis strain, whereas mutants bearing both SPI-1 and SPI-2 are almost as virulent as the wild-type strain (Rychlik et al. 2009). The role of SPI-1 T3SS has also been shown with *sipD* and *iacP* (invasion associated ACP) mutants which were unable to colonize spleens, whereas the wild-type *S*. Enteritidis strain could, three days postinfection in chickens infected subcutaneously (Parker and Guard-Petter 2001). However, numerous other data have shown that the role of the T3SS-1 is controversial in chicken and especially for the intestinal translocation (see the section “In vivo evidences for a non-essential role of T3SS-1”).

Until recently, little was known about the infection mechanisms of *Salmonella* in the plant kingdom. Contaminated plants are nonetheless responsible for 25% of food poisoning outbreaks in the United States (Rangel et al. 2005). Generally, it was believed that *Salmonella* could survive on leaves. However, a growing body of evidence points to an active process in which *Salmonella* infects plant organs and uses them as a viable host or vector between animals (Golberg et al. 2011). This invasion process uses both SPI-1 and SPI-2-encoded apparatus because the proliferation rate in plant of *invA* and *prgH* (encoded by SPI-1) mutants and *ssaJ* and *ssaV* (encoded by SPI-2) mutants are lower than the wild-type *S*. Typhimurium strain (Schikora et al. 2011). Moreover, *Salmonella* actively suppresses plant defense mechanisms using the SPI-1 T3SS. Mutants defective in virulence factors induced, indeed, disease symptoms (tissue damage, oxidative burst, and pH changes) contrary to the wild-type *S*. Typhimurium strain (Schikora et al. 2011; Shirron and Yaron 2011). Involvement of the T3SS appears to depend on the *Salmonella* serotype, plant species, and even the cultivar. Barak et al. (2011) showed, for example, diverse resistant/susceptible phenotypes to *Salmonella* in different tomato cultivars.

### In vivo evidences for a non-essential role of T3SS-1

Although numerous studies demonstrate a role of T3SS-1 in host infection, recent evidence from bovine, chicken, and murine models suggests that *S*. Typhimurium and other serotypes can also cause infection in a SPI-1-independent manner (Coombes et al. 2005; Hapfelmeier et al. 2005; Desin et al. 2009). For example, Morgan et al. (2004) concluded that *S*. Typhimurium uses different strategies to colonize calves and chicks. They observed that T3SS-1 and T3SS-2 are required for efficient colonization of cattle, whereas disruption of these secretion systems only caused a minor defect in *S*. Typhimurium colonization of chicks. The role of SPI-1 T3SS in chicken infection remains unclear. Desin et al. (2009) showed, for example, that a *S*. Enteritidis ΔSPI-1 mutant was not impaired in the caecal colonization of 1-week-old chicks, whereas the deletion of this region caused a delay in systemic infection. Similar results were observed by Rychlik et al. (2009)*,* who showed that SPI-1 from *S*. Enteritidis was poorly involved in intestinal chick colonization, but was involved in internal organ colonization. Another argument for a nonessential role of the T3SS-1 in vivo was reported by Murray and Lee (2000) in a murine model of infection. They found that a *S*. Typhimurium strain, in which SPI-1 was entirely deleted, was recovered from spleens of infected mice at a frequency similar to that of its parental wild-type strain, indicating that *S*. Typhimurium did not require T3SS-1 genes to cross the intestinal epithelium and infect systemic tissues. Moreover, it has been reported that nonfunctional SPI-1 mutants, which render them severely deficient for invasion of polarized epithelial cells, retain their ability to invade M cells in a murine gut loop model, implying that intestinal invasion may involve other factors (Clark et al. 1996).

The large majority of the in vivo studies on T3SS-1 have been performed with broad host range serotypes (*S*. Typhimurium and *S*. Enteritidis), and only a few analyses have involved host-specific serotypes. The translocation mechanism of typhoidal serotypes from the gut to other organs in farm animals, thus remains elusive. For *S*. Gallinarum, which induces a typhoid-like disease, the molecular and cellular mechanisms of fowl typhoid are relatively poorly understood, but the 85-kb *S*. Gallinarum plasmid has been shown to be essential for virulence, whereas a functional SPI-1 T3SS is not required. In addition, unidentified gene(s) under the control of ppGpp has (have) been shown to be involved in internalization (Jones et al. 2001; Jeong et al. 2008).

Overall, numerous articles have reported that *Salmonella* strains impaired in their capacities to use their T3SS-1 can still colonize the intestinal tissue and induce different pathologies in different animals, whereas others demonstrate an in vivo role of this T3SS. Discrepancies that exist between studies could, in part, be due to the type of infection induced (systemic/enteric), the time analyzed postinoculation, the serotype used, the host resistance, and the immune status of the host. The differences could also be due to the mutant used. For example, Murray and Lee (2000) using a ΔSPI-1 mutant demonstrated that SPI-1 is not essential, but they also found that *S*. Typhimurium strains containing a mutation in *hilA* or *invG,* which are encoded by SPI-1, were recovered from the intestinal tissues and internal organs of mice at a lower frequency than their parental wild-type strain. One explanation for such results is that deletion of SPI-1 genes suppresses the *hilA* infection defect and allows *Salmonella* to colonize and infect their hosts in a SPI-1-independent manner (Murray and Lee 2000).

All these in vivo experimental studies support clinical observations. Indeed, some *Salmonella* Senftenberg and *Salmonella* Litchfield isolates carrying a deletion encompassing a vast segment of SPI-1 have been identified (Ginocchio et al. 1997), and some of them have been responsible for food-borne disease outbreaks, indicating that SPI-1 is not required for enteropathogenesis in humans (Hu et al. 2008; Li et al. 2011).

## Existence of Other Entry Mechanisms

To infect different hosts, and cause various diseases ranging from typhoid fever to gastroenteritis or to an asymptomatic carrier state, *Salmonella* needs to cross several barriers. Crossing these barriers and multiplying within the host require invasion of a large variety of phagocytic and nonphagocytic cells. Research on the invasion mechanisms has until recently focused on SPI-1 and SPI-2 due to their key roles in entry and intracellular multiplication within different cell types, in particular enterocyte cell lines (Agbor and McCormick 2011). However, the role of outer membrane proteins (OMP) as possible adhesion molecules and virulence factors has been demonstrated in different pathogenic bacteria. In *Salmonella,* the association of OMP with host cells is known to trigger a variety of biological events that include induction of innate and adaptative immune response (Galdiero et al. 2003). More recently, some *Salmonella* OMP, namely Rck and PagN, have been shown to be able to induce cell invasion (Heffernan et al. 1994; Rosselin et al. 2010).

### Role of the OMP Rck

Rck is a 19-kDa OMP encoded by the *rck* gene located on the large virulence plasmid which contributes to the expression of virulence genes like the *spvRABCD* (*Salmonella* plasmid virulence), *pef* (plasmid-encoded fimbriae), *srgA* (SdiA-regulated gene, putative disulphide bond oxidoreductase), or *mig-5* (macrophage-inducible gene coding for putative carbonic anhydrase) genes (Rychlik et al. 2006). Among the different serotypes harboring a virulence plasmid (i.e., Typhimurium, Enteritidis, Gallinarum, Pullorum, Dublin, Abortus-ovis, and Choleraesuis), only a few carries the *rck* gene (Buisan et al. 1994). It is highly conserved in most isolates of *S*. Enteritidis and *S*. Typhimurium (Futagawa-Saito et al. 2010), found in some isolates of *S*. Dublin, but was not detected on the virulence plasmid of *S*. Choleraesuis, *S*. Gallinarum, and *S*. Pullorum (Chu et al. 1999; Rychlik et al. 2006). This gene is therefore present in the serovars which are frequently associated with infections of both humans and farm animals, and which have the wider range of hosts.

This protein belongs to a family of five homologous OMP characterized in *Salmonella* (Rck and PagC), *Escherichia coli* (Lom), *Yersinia enterocolitica* (Ail), and *Enterobacter cloacae* (OmpX) (Heffernan et al. 1992). A role in complement resistance, cell attachment and invasion has been attributed to individual members of this family. Molecular analysis has shown that Rck is homologous to PagC and Ail with 53% and 42% identity, respectively, but both complement resistance and invasion phenotypes have been attributed to only Rck and Ail. However, these proteins do not exhibit homologous regions that could support this role (Heffernan et al. 1992).

Rck alone is able to promote adhesion and internalization of coated beads (Rosselin et al. 2010) or of noninvasive *E. coli* strains (Heffernan et al. 1994). Fourty-six amino acids of Rck were identified as being necessary and sufficient in this process. Their binding to the cell surface is inhibited by soluble Rck and induces discrete membrane rearrangements due to reprogramming of cell signaling (Rosselin et al. 2010). These findings demonstrate that Rck induces a Zipper-like entry mechanism supporting the fact that *Salmonella* is the first bacterium to be described as able to induce both Zipper and Trigger mechanisms for host cell invasion. This Zipper entry process requires protein tyrosine kinase activation and class I PI3 kinase, which activate Akt and the small GTPase Rac1 and Cdc42 (but not Rho), leading to activation of the Arp2/3 complex and actin polymerization (Mijouin et al. 2012). The cellular receptor of Rck required in this process, however, remains unknown.

In spite of the absence of Rck expression in usual laboratory culture conditions, its role in *Salmonella* invasion has been demonstrated in vitro after growing *Salmonella* in swarming culture conditions (Rosselin et al. 2010). However, its role in *Salmonella* pathogenesis is still poorly understood. The fact that Rck expression is dependent on SdiA, a LuxR homolog which is a quorum sensing regulator, suggests an intestinal role of this invasin (Ahmer et al. 1998; Michael et al. 2001). However, *Salmonella* cannot synthesize the acyl homoserine lactones (AHL) that allow SdiA activation, but can detect AHL signaling molecules of other microbes using SdiA. Surprisingly, AHL have not been found in the intestinal tract of healthy mammals, with the exception of the bovine rumen (Erickson et al. 2002). SdiA from *Salmonella* could be activated in mice whose intestinal flora contained the AHL-producing *Yersinia enterocolitica* strain (Dyszel et al. 2010) or in turtle carrying *Aeromonas hydrophila* (Smith et al. 2008). After co-infection of mice with two *S*. Typhimurium strains engineered to produce AHL, a *sdiA* mutant and a *sdiA*^+^ strain, it was shown that the constant activation of SdiA conferred a selective advantage to *Salmonella* (Dyszel et al. 2010). However, under physiological conditions, SdiA activation did not confer a fitness advantage for intestinal colonization, suggesting that even if SdiA activation is achieved, it is not always sufficient to induce the expression of the Rck regulon. This hypothesis is supported by the fact that *rck* is also regulated by an unidentified SdiA-independent system (Smith and Ahmer 2003). Moreover, in relation to its role in resistance to the complement-dependent bactericidal action, it is conceivable that Rck also plays a role in systemic infection.

### Role of the OMP PagN

PagN is a 26-kDa OMP, which displays similarities with the Hek and Tia adhesins/invasins of pathogenic *E. coli. pagN* is widely conserved in the *Salmonella* genus. All the subspecies of *S. enterica* and two strains of *S. bongori* represented in the *Salmonella* reference collection C (SARC) carry the *pagN* gene (Boyd et al. 1996). This gene was originally identified in a Tn*phoA* random-mutagenesis screen of *phoP*-activated *S*. Typhimurium genes (Belden and Miller 1994) and through the use of in vivo expression technology performed in BALB/c mice (Heithoff et al. 1997). These studies showed that *pagN* is a PhoP-activated gene and thus is not expressed in *S*. Typhimurium strains grown under typical laboratory culture conditions, but is maximally expressed intracellularly as observed using gene fusion (Conner et al. 1998) or microarray analysis (Eriksson et al. 2003). The function of PagN as an invasin is supported by the fact that PagN overexpressed in a noninvasive *E. coli* strain induced cell invasion (Lambert and Smith 2008). However, PagN-defective bacteria displayed a consistent two- to fivefold reduction in cell invasion when compared to the wild-type strain only after overnight culture in pH 5.8 minimal media, suggesting that PagN is induced in the intracellular compartment. To correlate the intracellular expression pattern of PagN to its role as an invasin, Lambert and Smith (2008) postulated that *Salmonella* exiting from epithelial cells or macrophages might have an optimal level of PagN expression. Thus, PagN might facilitate interactions between *Salmonella* and mammalian cells in specific conditions that do not allow SPI-1 expression (Lambert and Smith 2008). This hypothesis is supported by the low T3SS-1 expression detected inside macrophages at 4 h postinfection (Eriksson et al. 2003) but not by the more recent data showing that SPI-1 genes remained expressed or were even upregulated few hours after invasion into different cell types (Drecktrah et al. 2005; Hautefort et al. 2008).

The PagN protein interacts with cell surface heparin sulfate proteoglycans to invade the mammalian cell line CHO-K1 (Lambert and Smith 2009). However, because proteoglycans cannot transduce a signaling cascade, they might act as co-receptors for invasion and not as the receptor per se. More studies are necessary to identify the PagN receptor at the molecular level.

### Role of the hemolysin HlyE

HlyE is a pore-forming hemolysin that is encoded by SPI-18, a small 2.3 kb genomic island missing in *S*. Typhimurium, but present in *S*. Typhi and *S*. Paratyphi A. HlyE shares more than 90% identity with the 34 kDa *E. coli* HlyE (ClyA) hemolysin. *S*. Typhi hlyE mutants are impaired in their ability to invade HEp-2 cells, compared with their wild-type parental strain. Moreover, the heterologous expression of HlyE in *S*. Typhimurium improves the colonization of deep organs in mice, demonstrating that HlyE is a new virulence determinant (Fuentes et al. 2008). This result is consistent with those from other laboratories showing that pore-forming hemolysins play critical roles in invasion of eukaryotic cells (Strauss et al. 1997; Doran et al. 2002). However, the precise mechanism by which hemolysins enhance invasion of intracellular pathogens remains unknown. It is possible that these hemolysins are invasins, but they could also modulate the entry of bacteria by inducing changes in calcium flux as described for *Listeria monocytogenes* (Dramsi and Cossart 2003).

### Existence of unknown factors involved in *Salmonella* invasion

The different data mentioned above show that *Salmonella* has developed different strategies to invade cells. Currently, three invasion pathways have been described for the broad host range serotypes (T3SS-1, Rck, PagN). However, recent data have shown that other unknown entry routes may be used depending on the serotype, the host and the cell-type considered.

Rosselin et al. (2011) have demonstrated that a *S*. Enteritidis strain which does not express Rck, PagN, and the T3SS-1 is still able to invade fibroblasts, epithelial, and endothelial cells significantly. The relative degree of the unknown entry processes depends on the cell type and the cell line. Among the cell types tested, 3T3 fibroblasts and MA104 kidney epithelial cells are the most permissive to these mechanisms, allowing one bacterium out of three (33% of the invasion) to enter independently of T3SS-1, PagN, and Rck. In contrast, nonpolarized HT29 human enterocytes are not prone to the unknown entry mechanisms, as less than 4% of the internalized bacteria invade cells in the absence of these known invasion processes. This heterogeneity in invasion profiles demonstrates a cell specificity of the T3SS-1, Rck, PagN-independent mechanisms. Other results reinforce the idea that nonidentified invasion factors are involved during the entry of *S*. Typhimurium. Indeed, Aiastui et al. (2010) and Sorge et al. (2011) have shown that nonidentified invasion factors are involved during entry of *S*. Typhimurium strains lacking the T3SS-1 into rat and mouse fibroblasts or into human brain microvascular endothelial cells. The entry mechanism used by *Salmonella* into immortalized human foreskin fibroblasts seems different from those described for Rck and PagN, as none of the GTPases tested (Rac1, Cdc42, RhoA, or RhoG) was essential for invasion of these cells (Aiastui et al. 2010). Moreover, the *Salmonella* SPI-1 T3SS is not required to invade intestinal cells grown in three dimensions as an *invA S*. Typhimurium mutant unable to express the T3SS-1, invades 3-D HT-29 cells at similar levels to the wild-type strain (Radtke et al. 2010). To our knowledge Rck and PagN are not expressed in bacterial culture conditions used in these studies; therefore, these results also highlight the fact that *Salmonella* possesses noncharacterized invasion factors.

Certain indications about the nature of the unknown invasion mechanisms have been obtained. Indeed, a *Salmonella* mutant expressing none of the known invasion factors displayed both local and massive actin accumulations, as well as discrete and intense membrane rearrangements. These observations obtained using different cell types show that invasion factors other than PagN, Rck, and the T3SS-1 apparatus are able to induce either a Zipper or a Trigger mechanism (Rosselin et al. 2011). Moreover, Zipper-like entry processes have been observed with fibroblast, epithelial, and endothelial cells (Aiastui et al. 2010; Rosselin et al. 2011; Sorge et al. 2011).

Overall and contrary to the prevailing theory, *Salmonella* can enter cells through a Zipper-like mechanism mediated by Rck and other unknown invasins, in addition to the Trigger mechanism mediated by its T3SS-1 apparatus and also other unknown determinants. These observations thus open new avenues for the identification of new invasion factors.

### Possible invasion factors

The cell specificity and consequently the organs or hosts targeted by *Salmonella* serotypes could be determined by the cells targeted by the different entry processes, in particular, by the Zipper mechanisms which involve a cell receptor. However, in addition to these different entry processes, the role of adhesins in cell specificity and invasion should also be taken into account. Fimbriae and/or nonfimbrial adhesins may indeed mediate attachment to cell surfaces, and thus could mediate part of the cell specificity. Moreover, some “typical” adhesins in some species are known to be involved, in other species, in entry in professional phagocytes or in nonprofessional phagocytes. This has been clearly described for *E. coli* where FimH mediates not only bacterial adherence but also invasion of numerous epithelial cells (Martinez et al. 2000).

Depending on the serotype, *Salmonella* can express a wide range of adhesion factors. *Salmonella* gene clusters encode more than 13 different fimbrial adhesins, such as Fim (type I fimbriae), Lpf (long polar fimbriae), Tafi (thin aggregative fimbriae, formerly SEF17), or the type IV pili of serotype Typhi (reviewed in Wagner and Hensel 2011). In addition, auto-transporter adhesins, such as ShdA, MisL, SadA, the type I secreted large repetitive adhesins SiiE, and BapA have been identified. Although the functions of various adhesins are not well understood, different studies have shown how they act in concert with other virulence determinants. For example, type 1 fimbrial adhesin FimH mediates binding to epithelial cells and it also helps to induce actin-dependent uptake in the absence of T3SS-1 in murine dendritic (Guo et al. 2007) or HeLa cells (Horiuchi et al. 1992; Lara-Tejero and Galan 2009). Similarly, Hensel's group has shown that SiiE mediates intimate contact of *Salmonella* with polarized cell surface allowing entry through the T3SS-1 (Gerlach et al. 2008). Mutant strains lacking SiiE fail to invade polarized cells. This giant nonfimbrial adhesin SiiE is secreted by a T1SS both encoded by the SPI-4. SPI-4 seems to be involved in calves but not in chickens or pigs challenged with *S*. Typhimurium (Morgan et al. 2004, 2007). The role of SPI-4 in virulence has also been demonstrated in mice following the oral challenge of BALB/c mice but not following the intraperitoneal infection of Nramp^r^ mice (Morgan et al. 2004; Lawley et al. 2006).

These adhesion factors could also be involved in cell specificity. To invade epithelial cells, *Salmonella* first binds to the cell surface. For instance, it is well known that some fimbrial adhesins play a role in targeting in vivo *S*. Typhimurium to a particular cell lineage in the host. For example, in an intestinal-organ culture model, the *pef* fimbrial operon mediates attachment to the murine villous small intestine, whereas selective adhesion of *S*. Typhimurium to murine ileal Peyer's patches is mediated by the *lpf* fimbrial operon. Similarly, MisL and ShdA are outer membrane fibronectin-binding proteins that are expressed in the intestine and could be involved in carrier state in mice (Kingsley et al. 2002; Dorsey et al. 2005). This synergy between adhesion and entry has been described for fimbriae and T3SS-1-independent internalization (Guo et al. 2007) and also for adhesins and T3SS-1 (Gerlach et al. 2008; Lara-Tejero and Galan 2009; Misselwitz et al. 2011).

The ability of some bacteria to invade human intestinal epithelial cells is also linked to outer membrane vesicles (OMV). It has been suggested that OMV carry effector proteins into the host cell resulting in the uptake of bacteria. In support of this hypothesis, purified *Treponema denticola* vesicles have been shown to harbor the chymotrypsin-like protease, dentilisin (PrtP), a protease that allows *T. denticola* to penetrate the epithelial barrier and Hep2 cells (Chi et al. 2003). In addition, a nonadherent-invasive *E. coli* mutant was discovered to have a defect in OMV formation (Rolhion et al. 2005). Recently, Kitagawa et al. (2010) have demonstrated that the OMP PagC is a major constituent of *Salmonella* OMV. However, until now, there has been no evidence of the role of these OMV in cell invasion by *Salmonella* because they could also function as a delivery system for virulence-related proteins from the SCV to the cytoplasm of macrophage cells (Kitagawa et al. 2010).

The T6SS represents a new paradigm of protein secretion that is crucial for the pathogenesis of many gram-negative bacteria. T6SS has been linked to a wide variety of functions ranging from inter-bacterial relationships, biofilm formation, cytotoxicity, survival in phagocytic cells, and also host cell invasion (Schwarz et al. 2010). Bioinformatic studies in different *S. enterica* serotypes have revealed four gene clusters encoding T6SS, acquired by independent lateral transfer events. These T6SS loci are located in different genomic islands: SPI-6, SPI-19, SPI-20, and SPI-21. The T6SS encoded in the SPI-19 is present in at least four serotypes: Dublin, Agona, Gallinarum, and Enteritidis. Interestingly, whereas *S*. Gallinarum appears to encode a complete T6SS, *S*. Enteritidis has a degenerate genetic element lacking most of the T6SS-related components (Blondel et al. 2009). SPI-19 contributes to the efficient colonization of the intestinal tract and systemic sites in chicks infected by *S*. Gallinarum strain 287/91 (Blondel et al. 2010). However, the transfer of a complete T6SS locus from *S*. Gallinarum to *S*. Enteritidis impaired the ability of this bacterium to efficiently colonize chicken tissues. The T6SS associated with SPI-6, formerly known as the *S. enterica* centisome seven islands (Sci), is present in at least 16 serotypes including Typhimurium and Typhi. Complete deletion of the Sci genomic island resulted in a decreased ability of *S*. Typhimurium to enter host cells (Folkesson et al. 2002). Similarly, increased levels of a dominant negative variant of *S*. Typhimurium ClpV strongly reduced the ability of this bacterium to invade epithelial cells (Schlieker et al. 2005). These results differ from the data obtained with a *sciS* (*icmF*-like) transposon mutant (Parsons and Heffron 2005). In that case, SciS limited intracellular growth in macrophages at late stages of infection, and attenuated the lethality of *S*. Typhimurium in a murine host. However, it is difficult to compare these studies due to the cell lines used (epithelial vs. macrophage). The function and characteristics of the T6SS are far from being understood, but this secretion system appears as a novel key player in bacterial pathogenesis and bacteria–host interaction.

In contrast, some bacterial invasion factors remain unidentified, whereas their cell receptors have been identified. Pier et al. found that human epithelial cells expressing wild-type CFTR (cystic fibrosis transmembrane conductance regulator**)** are significantly more infected by *S*. Typhi than cells expressing ΔF508 *Cftr*. Moreover, a 15-amino-acid peptide derived from the first CFTR extracellular domain inhibited *S*. Typhi invasion of T84 cells, whereas a scrambled synthetic peptide of these residues did not. These results indicate that CFTR is a major epithelial-cell receptor for *S*. Typhi internalization (Pier et al. 1998). In line with in vitro results, translocation of *S*. Typhi into the intestinal submucosa of ΔF508 *Cftr* heterozygotes was 86% less effective than in wild-type mice, and in homozygotes it was almost completely abrogated. In contrast, there was no significant difference between wild-type, heterozygous, or homozygous ΔF508 *Cftr* mice in intestinal barrier translocation of *S*. Typhimurium, demonstrating the specificity of the CFTR receptor for *S*. Typhi internalization (Pier et al. 1998).

## Conclusion

The precise traits associated with the zoonotic and epidemic potential of *Salmonella* strains remain unknown. This is particularly true with the recent identification of human clinical cases associated with SPI-1 deficient *Salmonella* strains indicating that SPI-1 T3SS is not required to cause entero-pathogenesis (Hu et al. 2008; Li et al. 2011). Identification of such traits is vital to assess the risk posed to humans by *S. enterica* strains found in animals or plants and for effective targeting of intervention strategies. Moreover, the reasons why some *Salmonella* serotypes are confined to the intestines while others translocate to internal organs remain unclear. A common feature of pathogens associated with enteric fevers is their use of different strategies to evade detection or subvert host innate immunity. Until recently, it was accepted that *Salmonella* entered cells only via its T3SS-1. However, new evidence has shown that *Salmonella* is able to use other pathways to enter phagocytic and nonphagocytic cells. Moreover, these T3SS-1-independent processes could mediate several Trigger and Zipper entry processes. We can thus speculate that these different entry mechanisms are efficient for particular cell types or species and could thus be involved in particular diseases induced by the different *Salmonella* serotypes. The coexistence of several invasion processes also raises the interesting possibility that synergy might exist between these different bacterial entry proteins or between the two invasion pathways (Zipper vs. Trigger) used by this pathogen. We can hypothesize that the receptor-ligand interaction mediated by the Zipper mechanism improves bacteria-cell contact and starts a process which facilitates the injection and the effects of bacterial effectors injected via the Trigger mechanism.

Several studies performed in different models suggest that the T3SS-1-independent entry processes might enhance bacterial entry into some cell lines or cell types of one or several species. They could also mediate invasion into polarized epithelial cells. Thus, particular invasins or entry processes may contribute to the invasion of specific host cells, and be involved in cell and tissue tropism as well as host specificity in both animals and plants. However, the number of identified adhesins or invasins which are only expressed in vivo is continually increasing, thus making it difficult to unravel the function of these new virulence factors. The use of more sophisticated in vivo studies, such as noninvasive in vivo imaging methods should help to understand the role of the entry factors in the interplay between bacteria and their hosts.

The new paradigm presented here stating that *Salmonella* strains are able to enter nonphagocytic cells by various routes should modify our view of the mechanisms that lead to the different *Salmonella*-induced diseases and should encourage us to revisit the host specificity bases. Future studies will undoubtedly focus on whether cell entry mechanisms are different according to the host and the serotype, and whether there is a link between host specificity, cell tropism, cell entry mechanism, cell response, and disease outcomes.
